# Cardiometabolic health and ASCVD primary prevention: time to take actions

**DOI:** 10.1038/s44325-025-00050-0

**Published:** 2025-05-03

**Authors:** Jing Liu

**Affiliations:** Department of Hypertension, Cardiometabolic Laboratory, Peking University People’s Hospital, Beijing, China

**Keywords:** Cardiology, Therapeutics, Dyslipidaemias, Hypertension, Endocrine system and metabolic diseases

## Abstract

Cardiometabolic disorders is highly prevalent worldwide. Curbing the disorders is critical for atherosclerotic cardiovascular disease (ASCVD) primary prevention. High blood pressure, cholesterol and glucose management, and lifestyle modification are essential for cardiovascular health. Sleep and mental health should also be addressed. *Life’s Vital 9*, a new concept for ASCVD primary prevention, is proposed. Actions should be taken to control cardiometabolic disorders and to facilitate the improvement of global cardiovascular health.

## Cardiometabolic disorders and CVD burden

Atherosclerotic cardiovascular disease (ASCVD) remains the leading cause of death worldwide. According to the recent Global Burden of Disease (GBD) report, cardiovascular disease (CVD) deaths increased about 50%, from 12.1 million in 1990 to 18.6 million in 2019, most of these deaths could be attributed to ASCVD^1^.

**Fig. 1:**
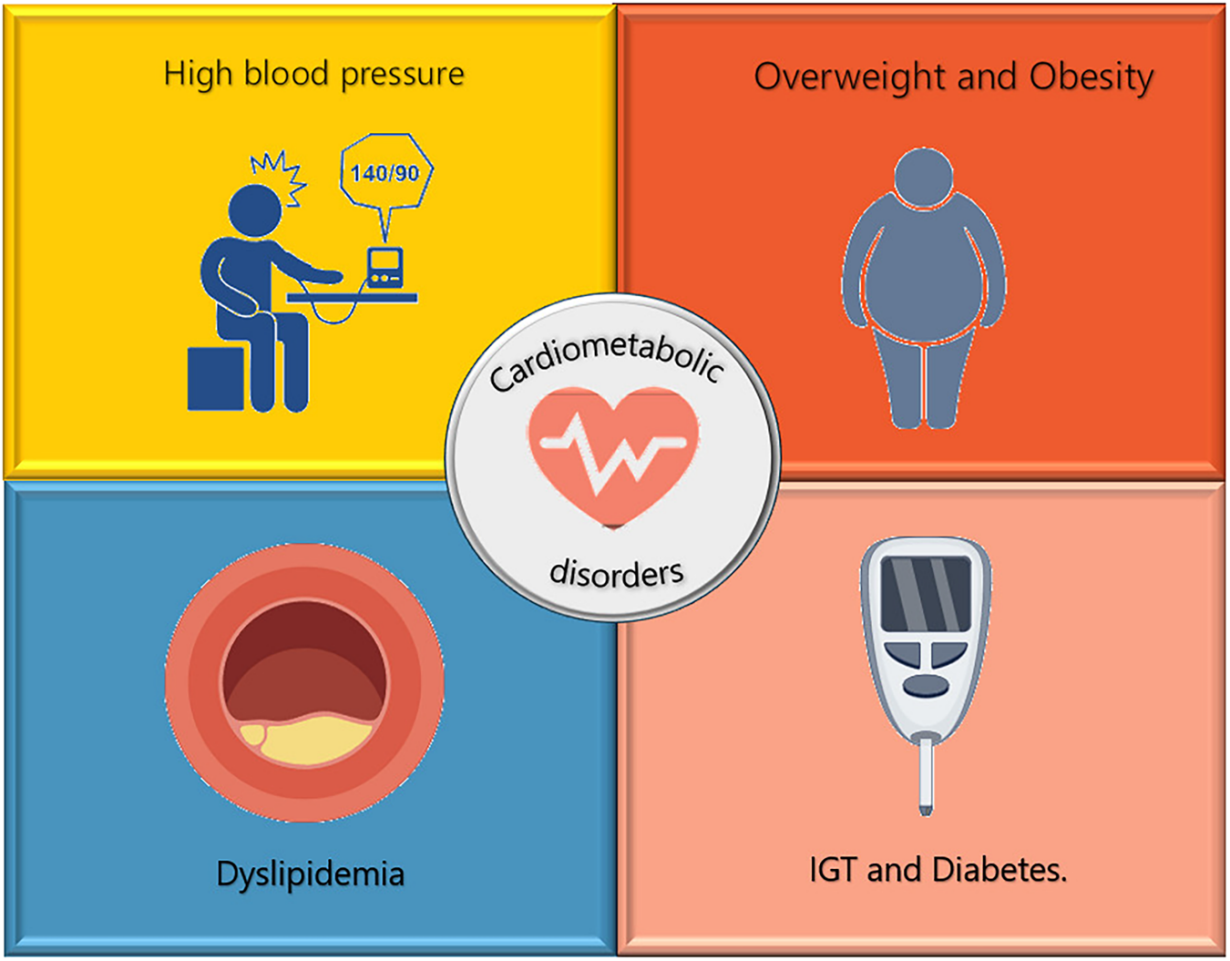
Cardiometabolic disorders. Cardiometabolic disorders, a constellation of overweight/obesity, high blood pressure, dyslipidemia and impaired glucose tolerance or diabetes.

Cardiometabolic disorders are a constellation of CVD risk factors, including overweight/obesity, high blood pressure (BP), dyslipidemia and impaired glucose tolerance or diabetes (Fig. 1). With population aging and the socioeconomic transition, alongside the sedentary lifestyle, the incidence of overweight/obesity and related cardiometabolic disorders has drastically increased in recent years. The global prevalence of obesity has increased by twofold and diabetes by fourfold over the past four decades, according to the Non-Communicable Diseases (NCD) Risk Factor Collaboration reports^2,3^. The global number of adults aged 30–79 years with hypertension doubled from 1990 to 2019, from 331 million women and 317 million men in 1990 to 626 million women and 652 million men in 2019^4^. Although non-optimal cholesterol and related deaths declined in high-income countries in northwestern Europe, north America from 1980 to 2018, but increased dramatically in low- and middle-income countries (LMICs) in east and southeast Asia and Oceania. In 2017, high non-HDL cholesterol was responsible for an estimated 3.9 million death worldwide, half of which occurred in east, southeast and south Asia^5^. Curbing the prevalence of the upstream risk factors is the key point for primary prevention of ASCVD^6^.

A healthy BP, blood glucose, cholesterol and adiposity are essential for cardiometabolic health. Unfortunately, an ideal cardiometabolic health was uncommon and a huge gap existed in reality, from west to east. Less than 7 percent of U.S. adults had optimal levels of cardiometabolic health components or free of CVD as of 2017–2018^7^. Large disparities still existed, as adults with less education were half as likely to have optimal cardiometabolic health compared with adults with more education, and Mexican Americans had one-third the optimal levels versus non-Hispanic white adults^7^. To define and improve cardiovascular health, a framework known as Life’s Simple 7 was proposed by the American Heart Association (AHA) in 2010. It refers to control common 7 cardiovascular risk factors, including non-smoking; body mass index (BMI) 18.5–24.9 kg/m²; moderate exercise ≥150 mins/week or vigorous exercise ≥75 mins/week; high intake of fruits, vegetables, whole grains, fish ≥2x/week, limit intake of sodium (<1500 mg/day), sugary drinks, and saturated fats; total cholesterol <200 mg/dL, BP < 120/80 mmHg, and fasting plasma glucose <100 mg/dL, that are modifiable through lifestyle changes^8^. It was estimated that ideal cardiovascular health was only 0.2% (0.1% in males and 0.4% in females) in Chinese adult population in 2010 and 1.7% in children and adolescents (1.9% in males and 1.6% in females) in 2013–2014^9,10^. There is a compelling need to take actions, from policy making, discipline construction to personal efforts to improve cardiovascular health throughout the world.

## Challenges at the policy level and potential solutions

**Fig. 2:**
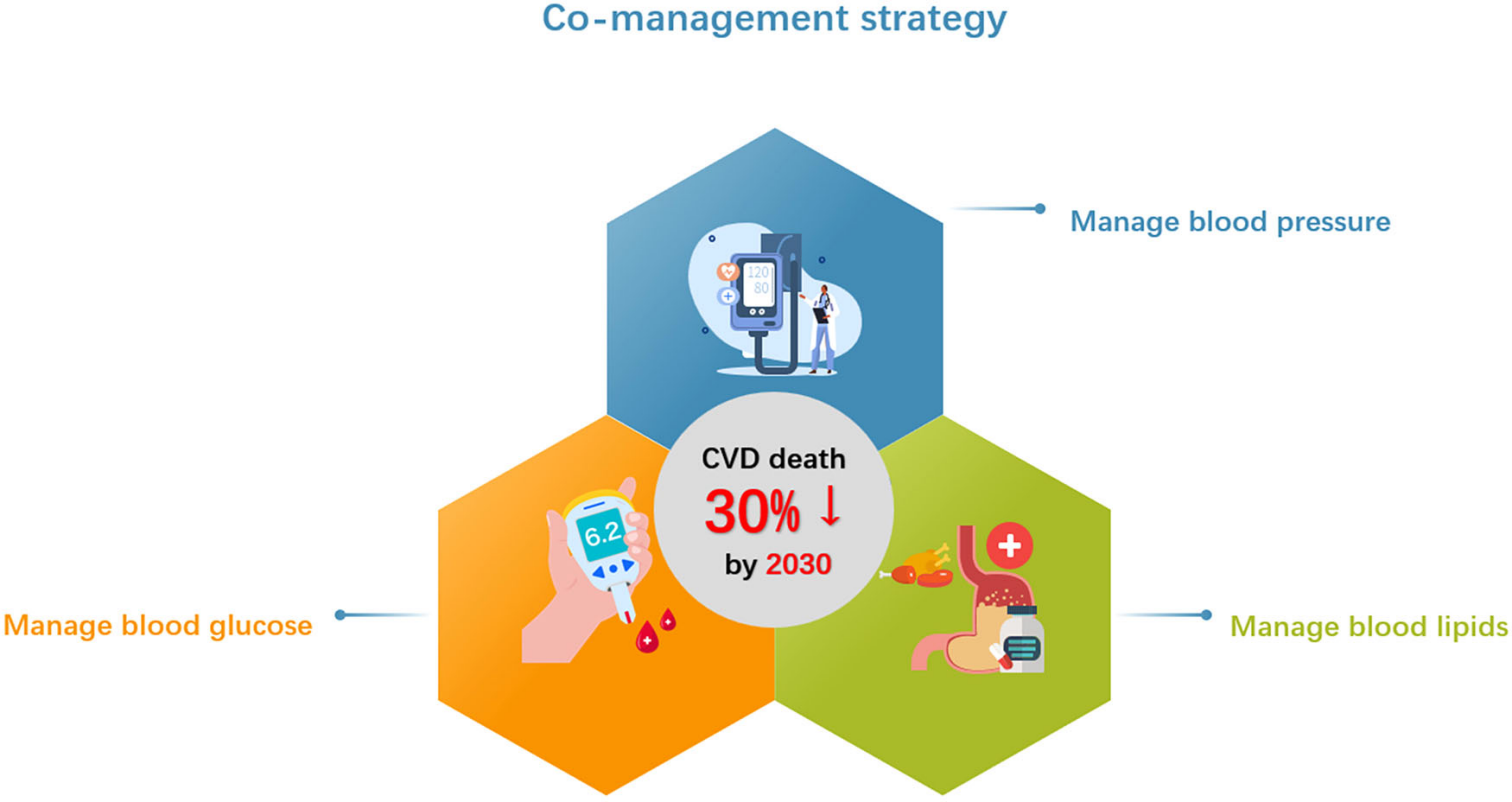
Co-management strategy for cardiometabolic disorders. Healthy China 2030, a pragmatic strategy of co-management of high blood pressure, high blood lipids and high blood glucose aiming for 30% reduction of cardiovascular death by 2030.

World Health Organization (WHO) first initiated the Health in the 2030 Agenda for Sustainable Development in 2016 to achieve a reduction of one third premature mortality from NCD through prevention and treatment by 2030^11^. To comply with the vision, population-based health initiatives have been proposed in different countries and entities. In China, an action of Healthy China 2030 has been launched since 2016, in which health is put on the priority list of development to a strategic position^12^. In a recent plan, a pragmatic strategy of co-management of high blood pressure, high blood lipids and high blood glucose was introduced to attain the goal of 30% reduction of CVD mortality by 2030^13^ (Fig. 2). In the U.S., Healthy People 2030, the fifth iteration of the Healthy People initiative with a data-driven national objectives to improve health and well-being over the next decade, was launched in 2020^14^. Both initiatives recognize cardiometabolic health as critical to national well-being and a multifaceted approach as solution, but adopt distinct approaches shaped by governance models, cultural contexts, and healthcare systems. China emphasizes structural reforms and traditional wellness, while the U.S. prioritizes equity, innovation, and localized solutions. Their progress offers valuable cross-learning opportunities for global health. Despite these efforts, challenges remain, as the mortality of coronary heart diseases and stroke in the U.S. are slightly increasing and far from the desired targets in 2021^15^.

Traditional designated disciplinary system is unfavorable for management of cardiometabolic disorders. Patients with these disorders have to refer to multiple disciplines, significantly increasing time and cost. An integrated modality for primary prevention is needed. Recently, a new concept of Cardiometabolic Medicine (CMM) has been proposed^16^. CMM integrates specialty of cardiology, endocrinology, internal medicine, etc., to establish a new model of patient-centered CVD prevention and risk management. To convey the concept of integration, CMM focuses on physician training and patient care. Specific fellowship training courses are designed for primary care or general cardiology physicians, the integrated thinking of management of cardiometabolic disorders would be established. A certification course on CMM is also optional for any interested clinician^17^. In patient-centric care approach, a multidisciplinary team, which covers clinical and preventive cardiology, hypertension, endocrinology, sports medicine, nutrition, sleep medicine and other specialties, is established to provide “one-stop” services and seamless management, from patient screening, to lifestyle intervention advice, to optimization of drug treatment, to rehabilitation, and to patient follow-up. This new model is expected to save medical resources, improve patient experience and help to promote harmonious doctor-patient relationship. Pilot centers have been established in Cleveland Clinic and other medical centers in US. AHA 2-year post-doctoral training program in cardiometabolic health has been initiated in Johns Hopkins University. Recent study demonstrated that the multidisciplinary patient-centric care approach was beneficial for overall CVD risk reduction^18^. Despite the theoretical advantages, it has to face the challenges in practice. Healthcare systems internal and external coordination, financial supports, and pragmatic training courses are needed.

## Specific pathways for personal behavioral interventions

Accordingly, as individuals should be made accountable for their own state of health, personal efforts on behavior modification with family and community support are unequivocally important for optimizing and preserving cardiovascular health.

**Fig. 3:**
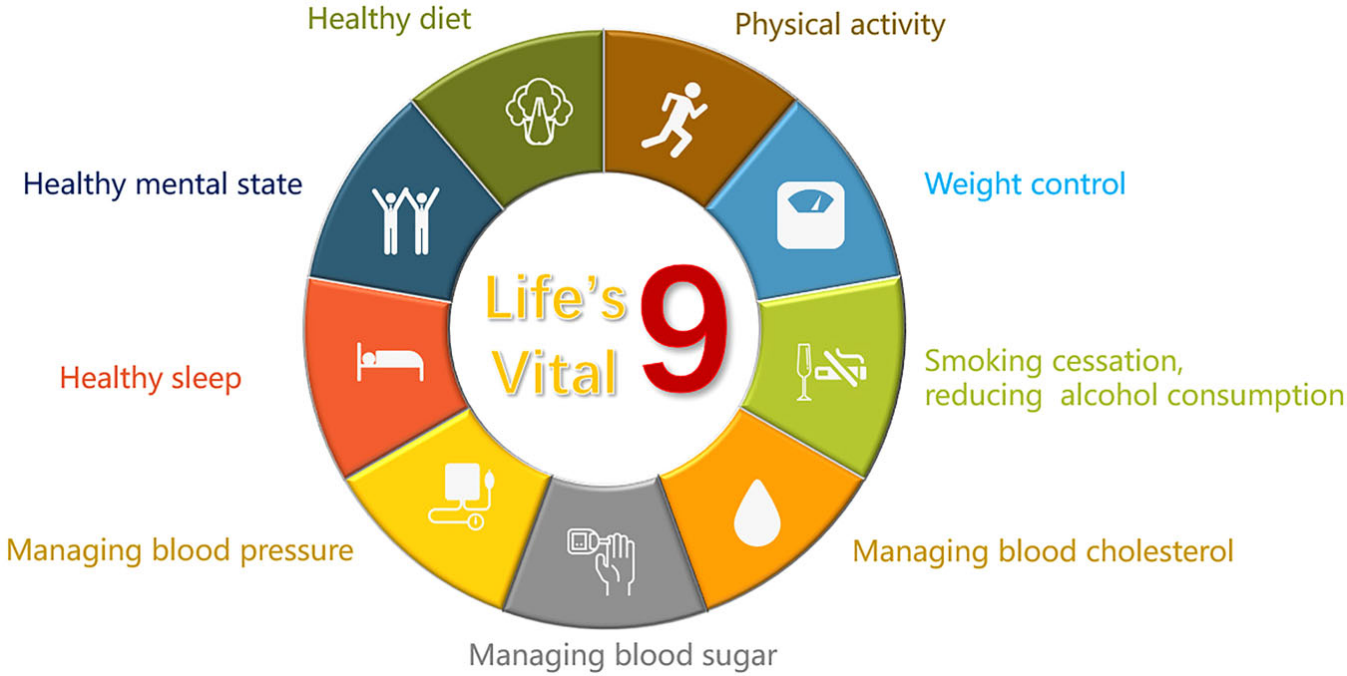
Life’s vital 9 for ASCVD primary prevention. Life’s vital 9, a novel health metrics for primary prevention of atherosclerotic cardiovascular disease (ASCVD).

Lifestyle modifications including restricted calorie and salt intake and low-fat/high-fiber diet, vigorous physical activity, weight control, smoking cessation and reduced alcohol consumption are of great value in maintaining cardiometabolic health and constitute the basement for CVD primary prevention^19,20^. Maintaining a healthy sleep with good-quality and adequate time is recommended in Chinese guidelines for primary prevention of CVD, as well as in the latest US Life’s Essential 8 metrics^20,21^. In addition, maintaining a healthy mental state should also be addressed. Mental and psychological abnormalities have impact on cardiovascular health, as evidence demonstrated depression and anxiety were related to the incidence of CVD and positive psychological well-being protected consistently against CVD, independently of traditional risk factors and ill-being^22^. Evidence-based approaches, including psychological counseling, meditation, or cognitive-behavioral therapy (CBT) can be adopted and incorporated into patient care accordingly. All above measures, together with healthy BP, glucose and lipids, constitutes the panorama of ASCVD primary prevention, here a new concept firstly proposed as *Life’s Vital 9* (Fig. 3).

Community programs can play a significant role in promoting cardiometabolic health. Regular physical activities, educational sessions on nutrition, fitness, smoking cessation and stress management can foster a supportive environment that encourages healthy lifestyles. Personalized health management tools are also essential. Mobile applications can track diet, exercise, and other lifestyle factors, offer meal plans that align with personal dietary needs and preferences or exercise routines that match one’s fitness level. Health metrics, such as oxygen saturation, heart rate and BP can be monitored via validated smart wearable devices (SWDs) when integrating with apps. Based on these data, tailored recommendations can be made to guide individuals towards a better cardiometabolic health.

## Intensive cardiometabolic management

Recently, a more aggressive strategy of management of hypertension, hypercholesterolemia and diabetes has been introduced for cardiometabolic health and CVD primary prevention. Optimal BP targeted below 130/80 mmHg with angiotensin-converting enzyme (ACE) inhibitors/angiotensin receptor blockers (ARBs)- based combination therapy, low density lipoprotein cholesterol less than 1.8 mmol/L on maximal tolerated statin with or without ezetimibe in patients at high risk of ASCVD, and new anti-diabetic medications including glucagon-like peptide type 1 receptor agonists (GLP-1RAs) and sodium glucose cotransporter 2 (SGLT 2) inhibitors in high risk diabetes, irrespective of blood glucose level, are recommended in primary prevention guidelines^19,20^. These measures fully reflect the concept of primary prevention of CVD via actively managing risk factors and promoting cardiometabolic health.

Despite the guidelines’ recommendation, treatment inertia is still one of the major barriers that block the translation of clinical evidence to practice in BP-, glucose- or lipid-lowering therapy in real world. Low patient adherence was commonly seen in the long-term treatment of cardiometabolic disorders, statins or BP-lowering medications were often reduced or even discontinued after treating to the target. Evidence demonstrated that polypill was a pragmatic solution for adherence improvement and CVD events prevention, as well as cardiometabolic management in LMICs^23^.

## Innovations and future technologies

Thanks to the innovations in science and technology, new medications and devices have been developed and used for management of cardiometabolic conditions. Non-alcoholic fatty liver disease (NAFLD), which is commonly comorbid with metabolic abnormalities including hyperlipidemia (69%), obesity (51%), hypertension (39%) and type 2 diabetes (23%)^24^, has been attractive for medication research and development. In recent randomized controlled trials, dual-acting glucose-dependent insulinotropic polypeptide (GIP)/GLP-1RA demonstrates advantage on weight loss as compared to GLP-1RA and placebo and holds promising on this emerging metabolic condition^25,26^. BP can be screened and monitored with SWDs. Continuous glucose monitors (CGM) have been used for diabetic patient management.

Telemedicine can provide remote consultant and real-time continuous cardiovascular health monitoring when integrated with SWDs, bridging the gap in healthcare access for patients in rural and underserved areas. At the same time, artificial intelligence (AI) is transforming cardiovascular health via improvement on diagnosis, treatment, and patient outcomes prediction. AI-powered SWDs (e.g., smartwatches) can continuously monitor heart rate, BP, and other vital signs, alerting patients and healthcare providers to potential issues. AI-integrated telemedicine platform can also provide cardiovascular health data analyzing and appointments scheduling, greatly increasing the accessibility of medical resources.

In addition, telemedicine platform and SWDs can be integrated with large language models (LLM) and deep learning algorithms, like ChatGPT and DeepSeek, which can power chatbots that handle initial patient inquiries, provide basic medical advice, personalized health tips and educational content based on their medical history and current health status, increase the interactivity between health professionals and patients. However, addressing challenges related to data quality and safety, regulatory compliance, user acceptance, and technical issues is crucial for its successful implementation. As AI technology continues to evolve, it can play pivotal roles in transforming new technologies, empowering management of cardiometabolic health and making healthcare more accessible and efficient.

## Health disparities and shared decision-making

Although tremendous improvement has been made on cardiovascular health in recent years, one fact that cannot be ignored is that health disparities still exist in different populations of the world. There is a pressing need to focus on implementing accessible and affordable interventions and a shared decision-making process that accounts for patients’ preferences, comorbidities, and ability on expenditure should be made for a better cardiometabolic health and primary prevention of ASCVD.

## Data Availability

No datasets were generated or analyzed during the current study.
